# Nasotracheal vs. Orotracheal Intubation and Post-extubation Airway Obstruction in Critically Ill Children: An Open-Label Randomized Controlled Trial

**DOI:** 10.3389/fped.2021.713516

**Published:** 2021-09-16

**Authors:** Vijay Kumar, Suresh Kumar Angurana, Arun Kumar Baranwal, Karthi Nallasamy

**Affiliations:** Division of Pediatric Critical Care, Department of Pediatrics, Advanced Pediatric Centre, Postgraduate Institute of Medical Education and Research, Chandigarh, India

**Keywords:** post-extubation stridor, extubation, airway edema, reintubation, post-extubation airway obstruction, nasotracheal intubation

## Abstract

**Background:** The data on long-term nasotracheal intubation among mechanically ventilated critically ill children is limited. The purpose of this study was to compare the rate of post-extubation airway obstruction (PEAO) with nasotracheal and orotracheal intubation.

**Methods:** This open-label randomized controlled trial was conducted in PICU of a tertiary care and teaching hospital in North India from January-December 2020 involving intubated children aged 3 months−12 years. After written informed consent, children were randomized into nasotracheal and orotracheal intubation groups. Post-extubation, modified Westley's croup score (mWCS) was used at 10-timepoints (0-min, 30 min, 1, 2, 3, 6, 12, 24, 36, and 48-h after extubation) to monitor for PEAO. The primary outcome was the rate of PEAO; and secondary outcomes were time taken for intubation, number of intubation attempts, complications during intubation, unplanned extubation, repeated intubations, tube malposition/displacement, endotracheal tube blockade, ventilator associated pneumonia, skin trauma, extubation failure/re-intubation, duration of PICU stay, and mortality.

**Results:** Seventy children were randomized into nasotracheal (*n* = 30) and orotracheal (*n* = 40) groups. Both the groups were similar in baseline characteristics. The rate of PEAO was similar between nasotracheal and orotracheal groups (10 vs. 20%, *p* = 0.14). The maximum mWCS and mWCS at 10-timepoints were similar in two groups. The time taken for intubation was significantly longer (85 vs. 48 s, *p* < 0.001) in nasotracheal group, whereas other secondary outcomes were similar in two groups.

**Conclusion:** The rate of PEAO was not different between nasotracheal and orotracheal groups.

**Clinical Trial Registration:**http://ctri.nic.in, Identifier: CTRI/2020/01/022988.

## Introduction

Endotracheal intubation is commonly performed intervention in critically ill children to provide mechanical ventilation in emergency rooms (ERs) and Pediatric intensive care units (PICUs). Orotracheal and nasotracheal intubation are two modes with their own advantages and disadvantages (1–3). Orotracheal intubation is generally preferred and commonly used as it is easier, quicker especially during emergent intubations, and less painful (1, 2). Nasotracheal intubation is commonly used in operating rooms especially during dental, oropharyngeal, and maxillofacial surgeries as it is easier to ventilate the patient and administer anesthetic gases without limiting access to oral cavity and oropharynx (1, 2). Nasotracheal intubation has several advantages as it is easier to secure; moves less, if secured properly; lesser risk of trauma to lips, tongue and larynx; lesser chances of unplanned extubation; more patient comfort; and possibly lower rate of post-extubation airway obstruction (PEAO) (1, 2). However, nasotracheal intubation can cause injury (to nose, turbinate, and nasopharynx), bleeding, and increases the risk of sinusitis (1, 2, 4–10). As nasotracheal intubation is technically challenging and associated with more complications, it is recommended that it should be performed by skilled healthcare providers (1, 2). Due to these reasons, nasotracheal intubation is less commonly practiced (2–5.6% of all endotracheal intubations) among adults and children undergoing mechanical ventilation in ICUs (2, 5, 11–15).

Few studies involving critically ill children on mechanical ventilation documented lower rate of unplanned extubation in nasotracheal group than in orotracheal intubation group (11, 16–18). However, the literature on the long-term nasotracheal intubation among mechanically ventilated critically ill children and its impact on PEAO is not available, despite the theoretical benefits of nasotracheal intubation. Therefore, we conducted this study to compare the nasotracheal and orotracheal routes of endotracheal intubation among mechanically ventilated critically ill children and compared the rate of PEAO between the two groups.

## Methodology

This open-label randomized controlled trial was conducted in a 15-bedded PICU of a tertiary care teaching hospital in North India over a period of 1 year (January 2020 to December 2020) including children aged 3 months−12 years with endotracheal intubation and invasive mechanical ventilation. The children with tracheostomy, raised intracranial pressure, severe acute respiratory distress syndrome (ARDS), refractory septic shock, remained intubated in ER for >24 h, referred intubated to ER from peripheral hospitals, anticipated intubation <24 h, cases requiring re-intubation after one episode of mechanical ventilation, known bleeding disorder, recent nasal surgery or trauma or burns, previous history of epistaxis, chronic lung disease, congenital heart disease, and with nasal and other facial malformation were excluded. The study protocol was approved by the Institute Ethics Committee (PGI/IEC/2019/002796, dated 28-12-2019) and registered with the Clinical Trials Registry-India (CTRI/2020/01/022988). The written informed consent was obtained from the parents/legal guardian before enrolment.

### Randomization

Patients were enrolled on the day of admission to PICU or whenever intubation was performed in PICU. The eligible children were randomized into 2 groups (nasotracheal orotracheal intubation groups) by using computer generated randomization table. The slips mentioning the group were placed in serially numbered, sealed, and opaque envelops which were opened at the time of randomization by the primary investigator (VK).

### Intubation Procedure

In our unit, we routinely perform orotracheal intubation. Children randomized to nasotracheal group were re-intubated through the nasal route. The primary investigator and senior residents working in the unit were trained in performing orotracheal and nasotracheal intubation. The standard protocol was followed to perform orotracheal and nasotracheal intubation. Adequate sedation, analgesia, and neuromuscular blockade (if needed) were used. Children were pre-oxygenated with bag and mask ventilation. The size (in mm) of endotracheal tube (ETT) was calculated as per the standard formulae for uncuffed (Age in years/4 + 4) and cuffed tube (Age in years/4 + 3.5). The length (in cm) of insertion of ETT was calculated as ETT size x 3 or Age in years/2 + 12 for orotracheal intubation and Age in years/2 + 15 for nasotracheal intubation (1, 19). We used micro-cuffed endotracheal tubes in all cases.

For nasotracheal intubation, the lidocaine jelly (as local anesthetic and lubricant) was applied to the nasal cavity and ETT prior to intubation. The ETT was then passed through nares into nasopharynx under direct laryngoscopy. Once it reached nasopharynx, it was guided into the glottic opening by using Magill's forceps (1, 2). During the procedure, oxygen saturation and heart rate was monitored continuously and time taken for intubation (in seconds) was recorded. The appropriate position of ETT was confirmed by clinical examination (auscultation over stomach and bilateral axilla) and later on by the chest radiograph, as per routine in the unit. The ETT was secured by the using dynaplast. For orotracheal intubation, one strip of dynaplast was pasted to the upper lip and another E-shaped strip was used to secure tube to upper and lower lip. For nasotracheal intubation, one strip of dynaplast was pasted to upper lip and another-Y-shaped strip was used to secure tube to upper lip. Any repositioning of the ETT after intubation was also documented.

### General Care

All children were managed and monitored as per unit's existing protocol for management of critically ill children for intubation, mechanical ventilation, sedation and analgesia, hemodynamic monitoring and treatment, nutrition, nursing support, weaning, extubation, and post-extubation care. Routine nursing care was provided in form of strict aseptic precautions, minimal handling, proper fixation of tube, clustering of interventions, and frequent position changes (if not contraindicated). The suction of endotracheal tube was done every 4–6-h or whenever needed. Enteral feeding was started as soon as possible, preferably within 24 h of admission to the PICU. Among children intubated for >48 h, six dosage of dexamethasone (0.5 mg/kg/dose) were used peri-extubation, with first dose given 24 h prior to extubation (20, 21). Feeding was withheld for 6 h prior to extubation and 4–6 h after extubation.

### Post-extubation Monitoring

We used modified Westley's croup score (mWCS) to monitor for PEAO at 10-timepoints (0-, 30-min, 1, 2, 3, 6, 12, 24, 36, and 48-h after extubation) (Supplementary Table 1) (20, 22, 23). A mWCS ≥4 suggested administration of adrenaline nebulization (1 mg/ml; 2.5 ml in 2.5 ml saline every 20 min until improvement). The re-intubation (by oronasal route) was performed if there was no response after adrenaline nebulization as evident by audible stridor, marked decreased air entry, severe chest indrawing and/or respiratory acidosis (pH < 7.35 and PaCO_2_ > 45 mmHg), SpO_2_ < 90% at FiO_2_ > 40%, bradycardia, or other clinical sign of impending respiratory failure, or mWCS of 7 (extubation failure) (20).

### Data Collection

Baseline data (age, sex, diagnosis), admission Glasgow Coma Scale (GCS), pediatric risk of mortality III (PRISM III) score, maximum vasoactive inotropic score (VIS), and sequential organ failure assessment (SOFA) score on day 1, 2, and 7 were noted. Time taken for intubation, number of intubation attempts, complications during intubation (hypoxemia, bradycardia, hypotension or cardiac arrest), unplanned or accidental extubation, repeated intubations, tube malposition or displacement, ETT blockade, skin trauma related to ETT, epistaxis, sinusitis, healthcare associated infections (HCAIs), ventilator associated pneumonia (VAP), post-extubation atelectasis, extubation failure/reintubation, duration of intubation, duration of PICU stay, and final outcome (survival or death) were recorded.

### Definitions

The clinically significant *PEAO* was defined as mWCS ≥ 4. *Time taken for intubation* was defined as period from stopping the bag and mask ventilation to starting positive pressure ventilation after insertion of ETT. *Intubation failure* was considered if there was bradycardia (heart rate < 60/min) and/or desaturation (SPO_2_ < 90%) or both during the intubation attempt. *Numbers of intubation attempts* defined as number of times procedure was aborted and requiring re-oxygenation and another attempt to intubate. The standard definitions were used for *Sepsis and severe sepsis* (24), and *VAP* (25). The skin trauma related to ETT was classified as per the standardized classification of decubitus lesions by the US National Pressure Ulcer Advisory Panel (NPUAP) (26).

### Outcomes

The primary outcome was the rate of PEAO among children in nasotracheal and orotracheal groups. Secondary outcomes were time taken for intubation, number of intubation attempts, complications during intubation, unplanned extubation, repeated intubations, tube malposition/displacement, ETT blockade, VAP, skin trauma related to ETT (injury to skin, nostrils, nasal septum, lip, or tongue), extubation failure/reintubation, duration of PICU stay, and survival or death.

## Data Entry and Statistical Analysis

The sample size was calculated based on the incidence of PEAO (32.8–34%) documented in previous studies from our PICU (21, 23). As a superiority trial, with the incidence of PEAO in nasotracheal group as 15% and β-error of 0.2, the required sample size was 90 cases in each group (*n* = 180).

However, in view of COVID-19 situation, the number of admissions to the PICU and those underwent mechanical ventilation were reduced (585 admissions during study period as compared to 900-100 admissions per year in normal times), leading to slower recruitment. The study was stopped after the end of the study period (with enrolment of 70 cases) as it was a dissertation project of a Pediatric Critical Care Fellow (VK), and the dissertations are time bound in our institute. The Dean of the institute approved to go ahead with the sample size of 70 (letter no. 12396/1TRG/PG-2019/15029, dated 16/12/2020).

Data entry and statistical analysis were performed using Microsoft Excel 2013 (Microsoft, Redmond, WA) and SPSS software version 21(IBM Corp. 2012. IBM SPSS Statistics for Windows, Version 21.0. Armonk, NY: IBM Corp). Descriptive statistics [mean (SD), median (IQR), range, number, and percentages] were used for baseline variables. Dichotomous outcomes were compared by chi-square test or Fisher's exact-test, as applicable. Continuous variables were compared by Student *t*-test or Mann–Whitney *U*-test. The repeated measure analysis of variance (RM-ANOVA) was used to compare mWCS between 2 groups over 10-timepoints. All tests used were two-tailed and *p*-value < 0.05 was taken as significant.

## Results

**Figure 1:**
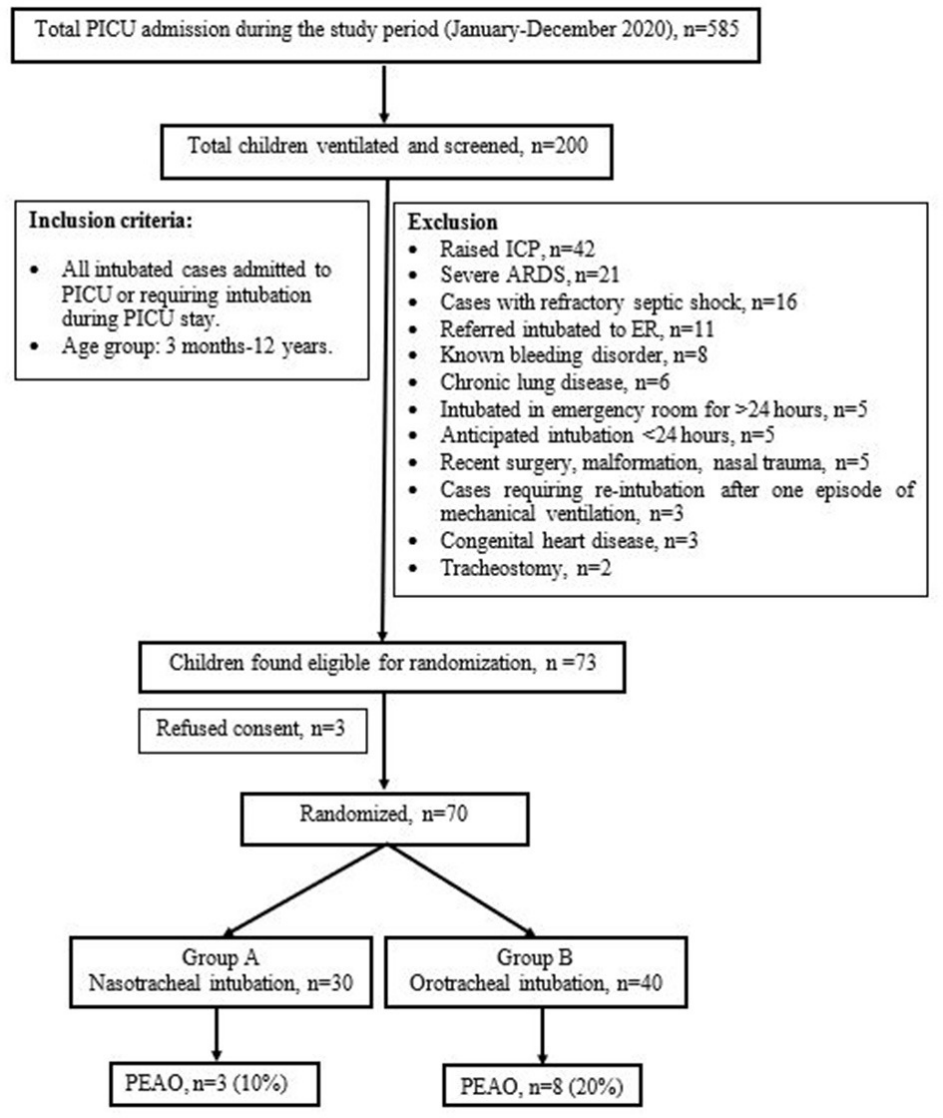
Study flow diagram.

During the study period, there were 585 admissions to the PICU, 200 (34.2%) were ventilated, and 70 were randomized to nasotracheal (*n* = 30) and orotracheal (*n* = 40) groups (Figure 1).

### Baseline Characteristics

There were 58.7% (*n* = 41) males with median (IQR) age of 36 (12–96) months. The most common diagnosis included snake envenomation (18.6%), metabolic disorder (12.9%), sepsis (11.4%), Landry-Guillain-Barre syndrome (11.4%), central nervous system infections (10%), Scrub typhus (10%), poisoning (10%), and ARDS (7.1%). The initial orotracheal intubation was performed in ER among 85.7% cases and in PICU among 14.3% cases. Later, the cases in nasotracheal group were extubated and reintubated through the nasal route. The median GCS at admission was 8 (6–10), PRISM-III score was 13 (9–20), and maximum VIS score was 50 (20–62). The SOFA score on day 1, 2, and 7 were 6 (4–9), 3 (2–8.5), and 1 (0–2), respectively. Both the groups were comparable as far as baseline variables are concerned. However, children in nasotracheal group had lower GCS at admission (*p* = 0.02) (Table 1).

**Table 1:** Baseline characteristics and severity scores among children in nasotracheal and orotracheal intubation groups.

**Patient characteristics**	**Total**	**Nasotracheal**	**Orotracheal**	***P***
	**(*n* = 70)**	**(*n* = 30)**	**(*n* = 40)**	
Male, *n* (%)	41 (58.6)	19 (63)	22 (55)	0.48
Age (month); median (IQR)	36 (12–96)	42 (21–133)	30 (11–85)	0.73
Diagnosis				
Snake envenomation, *n* (%)	13 (18.6)	9 (30)	4 (10)	0.12
Metabolic disorders, *n* (%)	9 (12.9)	4 (13.3)	5 (12.5)	
Sepsis, *n* (%)	8 (11.4)	2 (6.7)	6 (15)	
LGBS, *n* (%)	8 (11.4)	3 (10)	5 (12.5)	
CNS infections, *n* (%)	7 (10)	3 (10)	4 (10)	
Scrub typhus, *n* (%)	7 (10)	2 (6.7)	5 (12.5)	
Poisoning, *n* (%)	7 (10)	3 (10)	4 (10)	
ARDS, *n* (%)	5 (7.1)	1 (3.3)	4 (10)	
Disseminated Staphylococcal sepsis, *n* (%)	2 (2.9)	0	2 (5)	
Electrocution, *n* (%)	2 (2.9)	1 (3.3)	1 (2.5)	
Dengue shock syndrome, *n* (%)	1 (1.4)	1 (3.3)	0	
Tetanus, *n* (%)	1 (1.4)	1 (3.3)	0	
Site of intubation prior to enrolment				0.84
ER, *n* (%)	60 (85.7)	26 (86.7)	34 (85)	
PICU, *n* (%)	10 (14.3)	4 (13.3)	6 (15)	
GCS at admission, median (IQR)	8 (6–10)	7 (5–10)	9 (8–12)	0.02
PRISM III Score, median (IQR)	13 (9–20)	12 (9–15)	16 (11–20)	0.09
Maximum VIS score, median (IQR)	50 (20–62)	43 (13–53)	50 (21–65)	0.73
SOFA score, median (IQR)				
Day 1	6 (4–9)	5 (2–6)	8 (4–9)	0.07
Day 2	3 (2–8.5)	3 (2–4.2)	4 (2–9)	0.07
Day 7	1 (0–2)	1 (0–2)	1 (0–5)	0.42

### Primary Outcome

**Figure 2:**
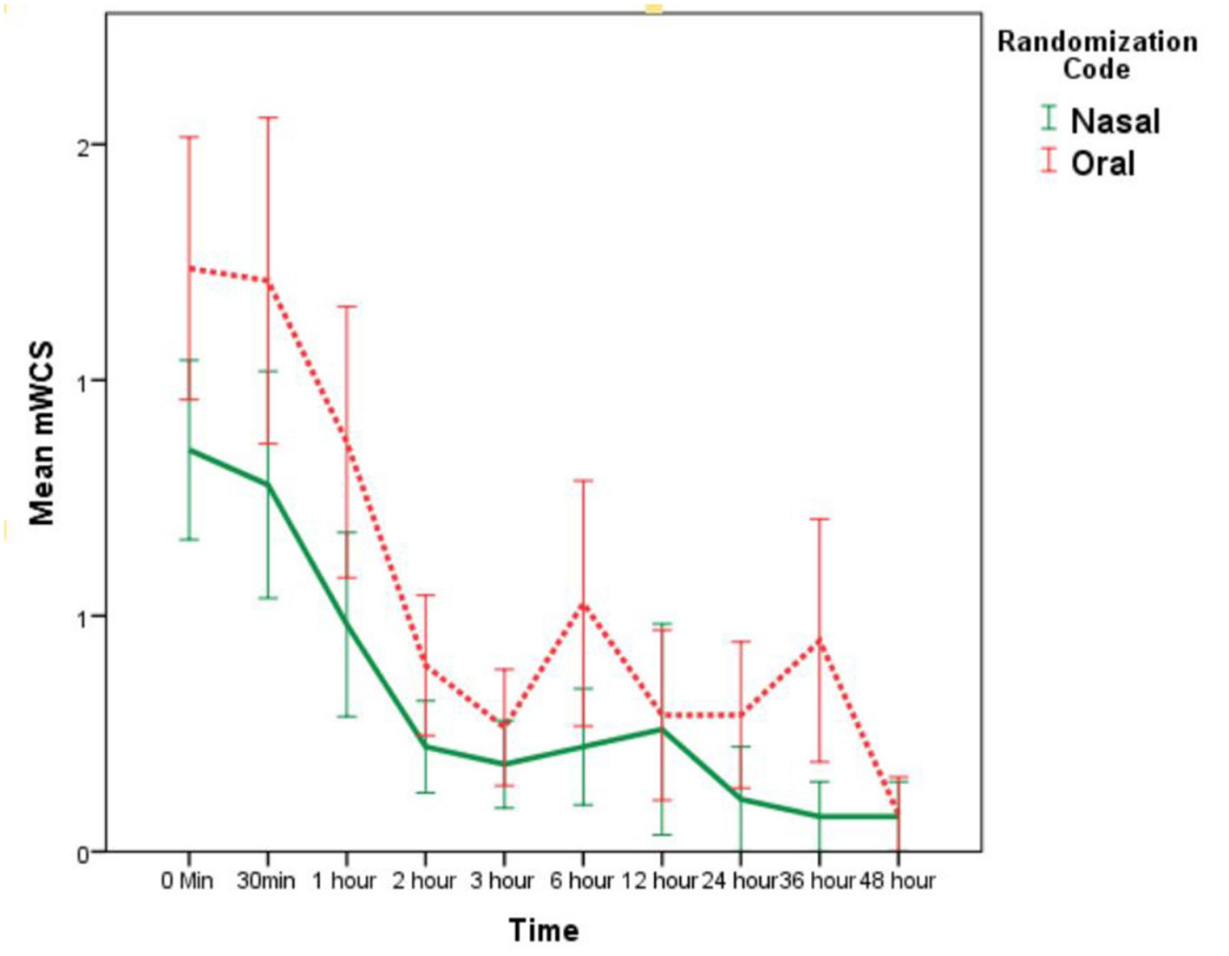
mWCS [mean (SE)] in nasotracheal and orotracheal intubation groups at different time points after extubation.

The overall rate of PEAO was 15% (*n* = 11). The rate of PEAO in nasotracheal and orotracheal groups was 10% (*n* = 3) and 20% (*n* = 8), respectively (*p* = 0.14). The maximum mWCS [mean (SE)] was 1.81 (0.25) and it was comparable in nasotracheal and orotracheal groups [1.62 (0.38) vs. 1.98 (0.33), respectively, *p* = 0.47] (Table 2). The serial mWCS in the first 48 h following extubation was also similar in two groups (Table 2; Figure 2). The RM-ANOVA showed no significant difference in mWCS between 2 groups over 10-timepoints (*p* = 0.53, Wilks Lambda Test).

**Table 2:** Primary outcomes in nasotracheal and orotracheal intubation groups.

**Outcome parameter**	**Total**	**Nasotracheal**	**Orotracheal**	***P-*value**
	**(*n* = 70)**	**(*n* = 30)**	**(*n* = 40)**	
Post-extubation airway obstruction, n (%)	11 (15.7)	3 (10)	8 (20)	0.14
Maximum Westley Croup Score (m WCS), mean (SE)	1.81 (0.25)	1.62 (0.38)	1.98 (0.33)	0.47
**WCS, mean (SE)**				
0 min	1.17 (0.18)	0.96 (0.21)	1.32 (0.27)	0.32
30 min	1.16 (0.22)	0.97 (0.24)	1.3 (0.33)	0.45
1 h	1 (0.23)	0.87 (0.31)	1.1 (0.03)	0.61
2 h	0.48 (0.14)	0.38 (0.16)	0.56 (0.22)	0.53
3 h	0.23 (0.09)	0.27 (0.13)	0.26 (0.12)	0.94
6 h	0.49 (0.18)	0.44 (0.26)	0.52 (0.26)	0.83
12 h	0.27 (0.13)	0.25 (0.22)	0.29 (0.18)	0.88
24 h	0.21 (0.1)	0.13 (0.11)	0.29 (0.16)	0.37
36 h	0.29 (0.15)	0.07 (0.07)	0.45 (0.25)	0.23
48 h	0.13 (0.79)	0.21 (0.05)	0.08 (0.03)	0.48

### Secondary Outcomes

The overall time taken [median (IQR)] for intubation was 60 (47–85) s and it was significantly higher in nasotracheal group as compared to orotracheal group [85 (75–90) s vs. 48 (45–60) s, respectively, *p* < 0.001]. Other outcomes like children requiring >1 intubation attempt (10 vs. 2.5%), complications during intubation (3.3 vs. 2.5%), unplanned extubation (10 vs. 15%), repeated intubation (10 vs. 15%), tube malposition/displacement (6.7 vs. 5%), ETT blockade (0 vs. 7.5%), skin trauma (10 vs. 5%), VAP (6.7 vs. 5%), duration of intubation (6.5 vs. 7 days), adrenaline nebulization (10 vs. 20%), post-extubation atelctasis (10 vs. 0%), type of post-extubation respiratory support, extubation failure/reintubation (6.7 vs. 8.5), duration of PICU stay (7.5 vs. 9 days), and mortality (6.7 vs. 12.5%) were similar in two groups (Table 3). The time of onset of PEAO in two groups was also similar (*p* = 0.22, Log Rank test).

**Table 3:** Secondary and final outcomes in nasotracheal and orotracheal intubation groups.

**Outcome parameter**	**Total**	**Nasotracheal**	**Orotracheal**	***P-*value**
	**(*n* = 70)**	**(*n* = 30)**	**(*n* = 40)**	
Time taken for intubation (seconds); median (IQR)	60 (47–85)	85 (75–90)	48 (45–60)	<0.001
Intubation attempts >1, *n* (%)	4 (5.7)	3 (10)	1 (2.5)	0.18
Complication during Intubation	2 (2.8)	1 (3.3)	1 (2.5)	0.84
Hypoxia/Bradycardia, *n* (%)				
Unplanned extubation, *n* (%)	9 (12)	3 (10)	6 (15)	0.54
Repeated intubation, *n* (%)	9 (12)	3 (10)	6 (15)	0.28
Tube malposition/displacement, *n* (%)	4 (5.7)	2 (6.7)	2 (5)	0.77
Endotracheal tube blockade, *n* (%)	3 (4.2)	0 (0)	3 (7.5)	0.13
Skin trauma related to ETT, *n* (%)	5 (7.1)	3 (10)	2 (5)	0.42
VAP, *n* (%)	3 (4.3)	2 (6.7)	1 (5)	0.14
Duration of intubation, median (IQR)	7 (3–13)	6.5 (3–13)	7 (3–13)	0.81
Adrenaline nebulization, *n* (%)	11 (15.7)	3 (10)	8 (20)	0.14
Post-extubation atelectasis, *n* (%)	3 (4.3)	3 (10)	0 (0)	0.08
Post-extubation respiratory support				
Nasal prongs, *n* (%)	14 (20)	6 (20)	8 (20)	0.62
Nasal CPAP, *n* (%)	29 (41.4)	15 (50)	14 (35)	
BiPAP, *n* (%)	13 (18.7)	5 (16.7)	8 (20)	
High flow nasal cannula, *n* (%)	2 (2.8)	1 (3.0)	1 (1.4)	
Extubation failure *n* (%)	5 (7.1)	2 (6.7)	3 (7.5)	1
Duration of PICU stay, median (IQR)	8 (5–13)	7.5 (4.7–14)	9 (5–13)	0.77
Death, *n* (%)	7 (10)	2 (6.7)	5 (12.5)	0.69

## Discussion

In this open-label RCT, we noted that in critically ill children undergoing mechanical ventilation, the rate of PEAO (10 vs. 20%) and maximum mWCS (1.62 vs. 1.98) were similar in nasotracheal and orotracheal intubation groups. The serial mWCS was also similar in two groups during the first 48 h after extubation. The rate of PEAO (15%) in the index study was within the range of the documented rates of PEAO among critically ill children (18–40%) (20, 23, 27–29). However, the rate of PEAO in index study was lower than the reported rates of PEAO in the recent studies from our unit (32.8–34%) (21, 23). The lower rate of PEAO could be due to the fact that we used micro-cuffed endotracheal tubes (high-volume-low-pressure) in all cases as these were routinely available from the hospital supply during the study period. The use of micro-cuffed ETT may had led to lesser movement of ETT, lesser chances of unplanned extubation or ETT change, lower risk of laryngeal edema, and hence lower rates of PEAO (15, 30, 31). None of the Pediatric studies looked into the impact of nasotracheal intubation on the rate of PEAO, time taken for intubation, unplanned extubation, extubation failure, and other important clinical outcomes (duration of PICU stay and mortality).

We noted that the nasotracheal intubation took more time than the orotracheal intubation, as it is technically more complex. However, other outcomes like children requiring >1 intubation attempt, complications during intubation, unplanned extubation, repeated intubation, tube malposition/displacement, ETT blockade, skin trauma, VAP, duration of intubation, adrenaline nebulization, post-extubation atelctasis, post-extubation respiratory support, extubation failure, duration of PICU stay, and mortality were similar in two groups. Previous studies also demonstrated that time taken for nasotracheal intubation was significantly longer than orotracheal intubation among critically ill adults and children (32–34). Also, nasotracheal intubation when compared to orotracheal intubation was associated with more changes in heart rate and blood pressure in early post-intubation period (33); need of more number of additional providers, more intubation attempts, and more traumatic intubations (34).

The literature on the outcome of long-term nasotracheal intubation in children on mechanical ventilation is limited. Spence and Barr (35) conducted a systematic review involving 2 randomized trials that compared nasal vs. oral intubation in neonates requiring mechanical ventilation and demonstrated that there were no differences between the orotracheal and nasotracheal route of intubation. One study noted higher rate of intubation failure using the nasal route; and one noted higher rates of post-extubation atelectasis in nasally intubated neonates weighing <1,500 g. The rates of ETT malposition, accidental extubation, tube blockage, re-intubation after extubation, septicemia, clinical infection, and local trauma were similar between two groups. Recently, Christian et al. (11) published a retrospective cohort study (January 2015 to December 2016) involving 121 PICUs in the United States and noted that 53% (*n* = 64) of PICUs had zero nasotracheal intubations during the study period. Out of 12,088 endotracheal intubations, only 5.6% (*n* = 680) were nasotracheal. Among nasotracheal group, the rate of unplanned extubation was significantly less as compared to orotracheal group (0.9 vs. 2.9%, *p* < 0.001). However. The rates of sinusitis and VAP were similar in two groups. Among children in nasotracheal group, majority were <2 years (88.1%), and 82.2% were classified as cardiac cases. Among young cardiac cases, the rate of unplanned extubation was significantly lower in nasotracheal group as compared to orotracheal group (0 vs. 2.1%, *p* < 0.001).

Unplanned extubation is one of the serious adverse events noted in cases with endotracheal intubation and associated with increased mortality, duration of mechanical ventilation, and ICU stay (16, 18, 36, 37). As ETT is well-secured with nasotracheal intubation, the chances of unplanned extubation are lesser, which has been demonstrated among adults and children (17, 38, 39). However, Piva et al. (40) demonstrated that among children in PICU, the rate of unplanned extubation was similar in orotracheal and nasotracheal group (3.1 vs. 1.6%, respectively, *p* = 0.06). Nasotracheal ETT can lead to blockage of drainage of paranasal sinuses, local trauma, edema, and local infection of nasal mucosa which can leads to sinusitis. The nasotracheal intubation has been identified as an important risk factor for sinusitis among adults and children (5–10). Moreover, the sinusitis can evolve into sepsis, bacteremia, and VAP (41). The rate of unplanned extubation was similar in two groups in the index study and none had sinusitis.

### Strength and Limitations

This is the first RCT that compared the nasotracheal vs. orotracheal route of endotracheal intubation in critically ill children receiving invasive mechanical ventilation. All enrolled cases were analyzed for the final outcome. We uniformly used micro-cuffed ETT in all cases. The limitations of this study include open-label trial as blinding of treating team and patients was not possible. The setting during endotracheal intubation was different in two groups, ER (in most cases) in orotracheal group and PICU in nasotracheal group, which is more of a controlled environment. In our units (ER and PICU), all cases underwent endotracheal intubation through orotracheal route first as per the routine practice. Children randomized to nasotracheal route were extubated and then re-intubation through nasal route. Hence, we could not enroll cases before endotracheal intubation and then randomizing them directly to orotracheal or nasotracheal groups. In nasotracheal group, the act of extubation and reintubation through nasal route at the time of enrolment can be a confounder as the number of airway manipulations may had a bearing on the occurrence of PEAO. The long-term outcome after discharge from the PICU was not available, as it was not the part of this study. We could enroll only 38.9% (70 out of 180) of the calculated sample size. To have an adequate answer to the study question, large randomized trial with adequate sample size is needed to assess the impact of nasotracheal intubation on PEAO, other clinical outcomes, and safety among children receiving long-term mechanical ventilation.

## Conclusion

In this open-label RCT involving critically ill children undergoing mechanical ventilation, we noted that the rate of PEAO was similar in nasotracheal and orotracheal intubation groups. Slower recruitment rate and enrolment of lesser than required sample size are the major limitations.

## Data Availability Statement

The raw data supporting the conclusions of this article will be made available by the authors, on reasonable request.

## Ethics Statement

The studies involving human participants were reviewed and approved by Institute Ethics Committee, PGIMER, Chandigarh, India. Written informed consent to participate in this study was provided by the participants' legal guardian/next of kin.

## Author Contributions

VK prepared the protocol, enrolled cases, collected data, reviewed the literature, and prepared the initial draft of the manuscript. SA conceptualized the study, supervised the preparation of the protocol and conduct of the study, analyzed data, and critically reviewed and finalized the manuscript. AKB supervised the data collection and helped in statistical analysis. KN supervised the data collection and performed literature review. All authors approved the final manuscript.

## Conflict of Interest

The authors declare that the research was conducted in the absence of any commercial or financial relationships that could be construed as a potential conflict of interest.

## Publisher's Note

All claims expressed in this article are solely those of the authors and do not necessarily represent those of their affiliated organizations, or those of the publisher, the editors and the reviewers. Any product that may be evaluated in this article, or claim that may be made by its manufacturer, is not guaranteed or endorsed by the publisher.
