# Supplementary material for: Nasotracheal vs. Orotracheal Intubation and Post-extubation Airway Obstruction in Critically Ill Children: An Open-Label Randomized Controlled Trial
Source: Front Pediatr. 2021 Sep 16;9:713516. doi: 10.3389/fped.2021.713516 (PMC8481700; doi:10.3389/fped.2021.713516)
Supplement: Supplementary file 1 [file Table_1.docx]

**Table 1: Modified Westley’s Croup Score (mWCS).**

| **Clinical Findings** | **Score** |
| --- | --- |
| **Inspiratory Stridor**   - None - Stridor audible with the stethoscope at rest - Stridor audible without stethoscope | 0  1  2 |
| **Air Movement**   - Normal - Decreased - Markedly decreased | 0  1  2 |
| **Retractions**   - None - Mild (Alar flaring) - Moderate (Suprasternal and intercostals) - Severe (all accessory muscles used) | 0  1  2  3 |
| Maximum Total Score  Need for adrenaline nebulization  Need for reintubation | 7  4  7 |
